# Standard protocol for the PIGRET assay, a high-throughput reticulocyte *Pig-a* assay with an immunomagnetic separation, used in the interlaboratory trial organized by the Mammalian Mutagenicity Study Group of the Japanese Environmental Mutagen and Genome Society

**DOI:** 10.1186/s41021-021-00181-7

**Published:** 2021-03-20

**Authors:** Satsuki Chikura, Takafumi Kimoto, Satoru Itoh, Hisakazu Sanada, Shigeharu Muto, Katsuyoshi Horibata

**Affiliations:** 1 Toxicology Research Department, Teijin Institute for Bio-medical Research, Teijin Pharma Limited, 4-3-2 Asahigaoka, Hino-shi, Tokyo, 191-8512 Japan; 2 Medicinal Safety Research Laboratories, Daiichi Sankyo Co., Ltd., 1-16-13, Kitakasai, Edogawa-ku, Tokyo, 134-8630 Japan; 3 Development ADMET Department, Translational Research Division, Chugai Pharmaceutical Co., Ltd., 1-135 Komakado, Gotemba-shi, 412-8513 Japan; 4 Safety Research Laboratories, Mitsubishi Tanabe Pharma Corporation, Shonan Health Innovation Park, 2-26-1, Muraoka-Higashi, Fujisawa, Kanagawa 251-8555 Japan; 5 Division of Genetics and Mutagenesis, National Institute of Health Sciences, 3-25-26 Tonomachi, Kawasaki-ku, Kawasaki-shi, Kanagawa 210-9501 Japan

**Keywords:** *Pig-a* assay, Glycosylphosphatidylinositol, Flow cytometry, Reticulocytes, In vivo gene mutation, CD59, HIS49, CD71, Immunomagnetics

## Abstract

The PIGRET assay is one of the *Pig-a* assays targeting reticulocytes (RETs), an in vivo genotoxicity evaluation method using flow cytometry with endogenous reporter glycosylphosphatidylinositol anchor protein. The PIGRET assay with RETs selectively enriched with anti-CD71 antibodies has several desirable features: high-throughput assay system, low background frequency of mutant cells, and early detection of mutation. To verify the potential and usefulness of the PIGRET assay for short-term testing, an interlaboratory trial involving 16 laboratories organized by the Mammalian Mutagenicity Study Group of the Japanese Environmental Mutagen and Genome Society was conducted. The collaborating laboratories assessed the mutagenicities of a total of 24 chemicals in rats using a single-treatment design and standard protocols for conducting the *Pig-a* assay on the total red blood cell assay and the PIGRET assay. Here the standard protocol for the PIGRET assay was described in detail.

## Background

The *Pig-a* assay is an in vivo gene mutation assay developed and validated in this decade [1–4]. An existing in vivo mutation assay with rodents requires transgenic animals (e.g., Muta Mouse, Big Blue mouse/rat, and *gpt* delta mouse/rat) [5]. However, the *Pig-a* assay can evaluate the mutagenicity of chemicals in wild-type rodents. It uses an endogenous reporter, phosphatidylinositol glycan class A gene (*Pig-a* in rodents and *PIG-A* in humans). The *Pig-a* gene is located on the X chromosome in mammalian cells [6, 7] and codes for an enzyme essential for synthesizing the glycosylphosphatidylinositol (GPI) anchor [6, 8–10]. Thus, once the *Pig-a* gene is disabled, GPI or GPI anchor proteins (e.g., CD59, CD55, and CD48 [11, 12]) are diminished on the cell surface and the cell is defined as a *Pig-a* mutant phenotype.

Since the *Pig-a* assays for rats and mice were reported in 2008 [13–16], various methods for the *Pig-a* assays with erythrocytes, leukocytes, or bone marrow cells have been developed. In 2013, a working group of the International Workshop on Genotoxicity Testing (IWGT) published consensus statements for the assays [17]. There are two major approaches have been utilized in the inter and intra laboratory trials (one is the Japanese group’s method and the other is a commercial kit, In vivo MutaFlow) for the *Pig-a* assays using rat erythrocytes from peripheral blood. In the IWGT workgroup report, both approaches have been concluded to be robust and reproducible on the available data. The Japanese research group conducted the *Pig-a* assay using the anti-erythroid marker antibodies (Abs; clone HIS49), including the red blood cell (RBC) *Pig-a* assay [18] and the PIGRET assay, in collaborative studies supported by the Japan Health Sciences Foundation (JHSF) [1, 19–23] and the Mammalian Mutagenicity Study Group of the Japanese Environmental Mutagen and Genome Society (MMS/JEMS). The PIGRET assay, the method described in this protocol, was developed in 2011 by Kimoto et al. [24]. The PIGRET assay uses immunomagnetic separated reticulocytes (RETs) from peripheral whole blood with PE-labeled anti-CD71 Abs. RETs selectively enriched with anti-CD71 Abs enable low spontaneous mutant frequency (MF) and high-throughput assay. In the PIGRET assay, spontaneous MFs are 5 × 10^− 6^ or below, which permits highly sensitive detection of mutagens. A quick response to the increase in MF is also a huge benefit for the PIGRET assay. RETs usually turn over in a couple of days and reflect the proliferation of mutant cells earlier than whole RBCs. In the MMS/JEMS collaborative studies across 16 laboratories, the PIGRET assay detected significant increases of MFs at 1 week after single dosing of typical mutagens [2]. The PIGRET assay counts more than 1 million cells (RETs) as an actual measuring number. The raw output data make it easy to determine whether a chemical of interest has mutagenic potential.

**Fig. 1:**
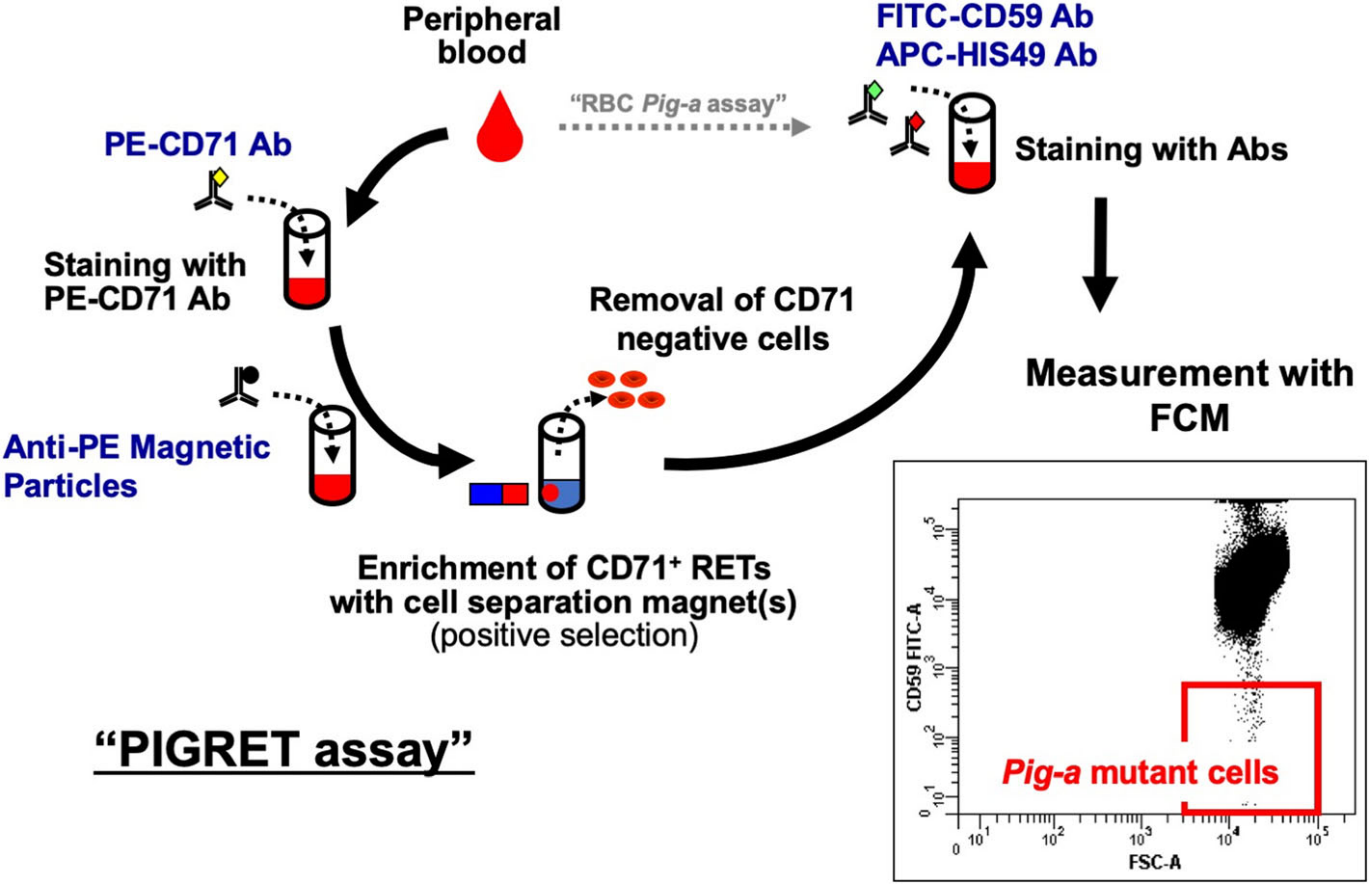
Outline of the PIGRET assay procedure. First, stain RBCs with PE-conjugated anti-CD71 Abs and mixed with anti-PE magnet particles. Enrich CD71 positive reticulocytes using cell separation magnet(s) by a positive selection method. Next, stain the enriched RETs with APC-conjugated anti-HIS49 Ab and FITC-conjugated anti-CD59 Ab. Finally, using FCM, CD71 and HIS49 double-positive cells are analyzed for CD59 expression, and *Pig-a* mutant cells are detected as the FITC-negative population

The *Pig-a* assay is listed in the International Council for Harmonisation of Technical Requirements for Pharmaceuticals for Human Use M7(R1) guideline as an in vivo follow-up study for positive in vitro findings [25]. The preparation for the new Organisation for Economic Co-operation and Development (OECD) test guideline for the in vivo *Pig-a* assay is ongoing [26, 27]. In this paper, a detailed procedure used in the MMS/JEMS collaborative study as a standard protocol was described for the PIGRET assay in rats. An outline of the assay is shown in Fig. 1. Peripheral blood is collected from rats and mixed with an anticoagulant. CD71-positive RETs are enriched from blood using the anti-PE-CD71 Ab and magnetic beads. Enriched RETs are stained with fluorescent-labeled anti-CD59 Ab and anti-erythroid marker Ab (clone HIS49), and then analyzed by a flow cytometer (FCM). At least 1 million RETs of each sample are analyzed to calculate the frequency of CD59-negative cells, which is defined as the *Pig-a* MF. In addition, whole blood cells are stained with HIS49 and anti-PE-CD71 Ab, and the ratio of RETs to the total RBCs (%RETs) is calculated using FCM.

## PIGRET assay standard protocol

### Instruments

The PIGRET assay uses FCM equipped with blue and red lasers and its corresponding analysis software. Here, we describe the standard procedure for the PIGRET assay with FACSCantoII (BD Biosciences) equipped with 488 nm blue and 633 nm red lasers and a FACSDiva software (BD Biosciences) for analysis as an example. A single-laser FCM can be used if an alternative fluorescent label (e.g., PerCP-Cy5.5) is used on an anti-rat erythroid Ab. Refer to the RBC *Pig-a* assay standard protocol and an article reported by Kikuzuki et al. [28]

For immunomagnetic separation, cell separation magnets, such as IMagnet Cell Separation Magnet (BD Biosciences), are required. Many kinds of cell separation magnets are commercially available, but they hold up only a small number of samples at one time. It would be better to use two or more magnets to proceed efficiently.

### Chemicals and materials

Anti-R-phycoerythrin (PE) Magnetic Particles-DM (clone E31–1459) and BD IMag buffer (10× buffer) are purchased from BD Biosciences. FITC-conjugated anti-rat CD59 Ab (FITC-CD59 Ab; clone TH9; BD Biosciences), APC-conjugated anti-rat erythroid marker Ab (APC-HIS49 Ab; clone HIS49; BD Biosciences), and PE-conjugated anti-rat CD71 Ab (PE-CD71 Ab; clone OX-26; BD Biosciences) are obtained commercially. Phosphate-buffered saline (PBS; Ca- and Mg-free) is required to dilute the blood samples and EDTA-2 K solution (12 mg/mL) is used as an anti-clotting regent for tail vein blood collection.

### Animals and dosing

Healthy young adult rats, usually 6–10 weeks old at the start of dosing, are used. Male or female rats can be used for this assay [29–31]. Groups of 6 rats are recommended at the 6th IWGT Workgroup meeting.

At least three test substance dosing groups and a negative/viechle control group should be used. Chose the route of administration to ensure adequate exposure of bone marrow, and generally the anticipated route of human exposure should first be considered. The maximum and lower doses should be selected according to the criteria given in the OECD Test Guidelines for in vivo genotoxicity and general toxicity studies [5, 32, 33].

A positive control group is required as an acceptance criterion to validate the PIGRET assay technique on-site. *N*-nitroso-*N*-ethylurea (Cas# 759–73-9; ENU) may be used as a positive control compound, and a single dose of 40 mg/kg ENU by gavage induces significant increases in *Pig-a* MF. Dissolve ENU in warm PBS (pH adjusted to 6.0–6.1, 37 °C) and filter the solution to remove undissolved particles. Keep it at room temperature away from light and use within 2 h after preparation.

### Blood collection and preservation

#### Blood collection

Blood is collected from rats before and at an appropriate time after the administration of the test compound. In the MMS/JEMS collaborative study, the PIGRET assay was conducted before and at 1, 2, and 4 weeks after a single administration.

Peripheral blood (80–150 μL) is collected from the tail vein and mixed well with 12 mg/mL EDTA-2 K at a ratio of 9:1 or 10:1. The collection volume was determined based on the animal’s age and the effects of bone marrow suppression due to the treated compound [see Note (a)]. When the test chemical is supposed to reduce %RET, the blood volume could be increased up to 150 μL or more per animal to obtain enough numbers of RETs. It is also possible to collect blood from the abdominal aorta using Vacutainer blood collection tubes containing anticoagulants [e.g., EDTA; see Note (b)]. Store blood samples on ice or in a refrigerator (2 °C–8 °C).

The coagulation of the blood samples has a negative impact on *Pig-a* assay data and may cause false-positive results due to poor staining. The blood sampling method is left to the operator’s discretion as long as blood coagulation is avoided.

#### Storage of blood samples

Blood samples in the refrigerator should be used for the *Pig-a* assay within 7 days of collection. Collection tubes are tightly capped and stored in a refrigerator (2 °C–8 °C). Tips for blood preservation were described in the RBC *Pig-a* assay standard protocol [18].

### Blood processing for the PIGRET assay

#### Immunomagnetic separation for CD71-positive cells

Two or more cell separation magnets may be helpful to proceed with this step. When the number of samples to be processed exceeds the number of slots in the prepared cell separation magnets, the samples are divided into two or more batches, and the procedure is followed [see Note (c)].

Prepare a premix solution containing 1 μg PE-CD71 Ab. Specifically, 5 μL PE-CD71 Ab stock solution (0.2 mg/mL) and 195 μL of PBS are mixed per sample. The total volume of the premix solution is determined to include the CD71 single-stain sample and some extras.

Dispense 200 μL of premix solution containing 1 μg PE-CD71 Ab into each sample tube, and add an appropriate volume (80–150 μL) of the blood sample [see Note (a)]. Mix well by pipetting or vortexing and incubate the samples for 15 min at a temperature of approximately 4 °C in the dark. After incubation, add 2 mL of IMag buffer diluted 10-fold with purified water to each sample tube and mix gently. Centrifuge the samples at approximately 1700×*g* for 5 min at room temperature and carefully remove the all supernatant while tilting the tube carefully.

Loosen the pellet by tapping and, then, add 50 μL PE Magnetic Particles Plus-DM (mix well by inversion or vortexing just prior to use) to each sample, and mix thoroughly with the blood cell pellet by pipetting approximately 10 times. Incubate at 6 °C to 12 °C (a general refrigerator could be used) for 15 min in the dark. Then, add 1 mL IMag buffer and mix by vortexing or pipetting until the pellets are completely dispensed. Immediately place the tube onto the cell separation magnet and incubate at room temperature for 6 min or longer. Aspirate the supernatant carefully with the tube placed on the magnet.

Remove the tubes from the magnet and add 1 mL IMag buffer to each tube. Disperse the pellet by pipetting and place the tubes to the magnet and incubate at room temperature for at least 2 min. Aspirate the supernatant on the magnet. Remove the sample tubes from the magnet and add 1 mL IMag Buffer to each tube again. Disperse the pellet by pipetting and put the tubes back onto the magnet and incubate at room temperature for another 2 min or longer. Completely remove the all supernatant with placed on the magnet.

Remove the sample tubes from the magnet and add 200 μL PBS. It is critical to thoroughly disperse the pellet at this point to keep the cells from clumping. Store the samples in the refrigerator until the other batch samples are ready.

#### Preparation of diluted APC-HIS49 Ab solution and master mix solution

Dilute the HIS49 Ab stock solution with PBS to 0.0667 mg/mL; specifically, dilute three-fold 0.2 mg/mL APC-HIS49 Ab stock solution with PBS. This diluted solution is used for master mix preparation and %RET analysis [see Note (d) for single-laser FCM].

One μg FITC-CD 59Ab and 0.133 μg APC-HIS49 Ab are combined per sample in the master mix solution. Specifically, 2 μL FITC-CD59 Ab stock solution (0.5 mg/mL) and 2 μL diluted APC-HIS49 Ab solution (0.0667 mg/mL) are mixed per sample. The total volume of the master mix solution is determined to include some extra and 4 μL of the master mix solution are used to stain each sample.

#### Staining assay samples

Add 4 μL of the master mix solution prepared above to fresh sample tubes, preventing any Ab from adhering to the tube wall. Add the whole volume of enriched CD71-positive sample (200 μL) to each tube and mix by pipetting several times. After adding the master mix solution to all samples, vortex the sample tubes again for several seconds. If enriched RETs splash on the upper part of the tube, mix well until the RETs are mixed with antibodies completely, or transfer the sample solution to a new tube.

Incubate samples at room temperature in the dark for 30 min. After incubation mix the samples by vortexing, and then centrifuge at approximately 1700×*g* for 5 min at room temperature. Remove the supernatant with an aspirator or a pipette while tilting the tubes.

Loosen the pellet by gently tapping the bottom of the tube, and add 0.5 mL or more PBS. Then, vortex the samples to thoroughly disperse the pellet. Adjust the total volume by additional PBS to attain the desired sample flow rate on the FCM; see the ‘Data collection’ section below. Keep the samples at room temperature in the dark until FCM analysis.

#### Staining for control samples for gate adjustment

A non-stained sample, a CD59 single-stain sample, and a HIS49 single-stain sample were prepared. For control samples, dispense 0.2 mL PBS into sample tubes, and take 3 μL blood collected from an animal in the negative or vehicle control group. Then, add 2 μL FITC-CD59 Ab stock solution (containing 1 μg Ab) to the CD59 single-stain sample tube, or add 2 μL diluted APC-HIS49 Ab (containing 0.133 μg Ab) to the HIS49 single-stain sample tube. Incubate the two single-stain samples and the non-stained sample for 30 min.

For the PIGRET assay, a CD71 single-stain sample is required. Add 3 μL blood to a new sample tube for the CD71 single-stain sample tube, and add 200 μL of the diluted PE-CD71 Ab premix solution (containing 1 μg PE-CD71 Ab) prepared in advance. Incubate the sample at a temperature of approximately 4 °C for 15 min in the dark.

Centrifuge all control samples at approximately 1700×*g* for 5 min at room temperature and then resuspend with about 1 mL PBS.

### Blood processing for %RET analysis

The procedure described here is for determining the ratio of RET (%RET) by FCM. This procedure defines RET as a CD71 and HIS49 double-positive cell. An automated hematology analyzer used for general toxicology tests, such as ADVIA (Siemens Healthineers) or equivalent, can also measure %RET. It is desirable to measure %RET in the same way throughout a time-course study.

Prepare the Ab master mix in the proportion of 2 μL diluted APC-HIS49 Ab (0.0667 mg/mL) and 5 μL PE-CD71 Ab stock solution (0.2 mg/mL) in 200 μL PBS per sample. The total volume of the mixture is prepared to include all samples and some extras.

Add 200 μL of the Ab master mix to new sample tubes, and then, add 3 μL blood to each tube. Mix the samples for several seconds and incubate at room temperature in the dark. After incubation, mix the samples again for several seconds and centrifuge at approximately 1700×*g* for 5 min at room temperature. Remove the supernatant with an aspirator, and the pellets by gentle tapping. Add about 1 mL PBS to each tube and resuspended. Store the samples at room temperature in the dark until FCM analysis.

### Flow cytometry

The following steps vary depending on the FCM model and analysis software. The procedures described below used FACSCantoII and FACSDiva software but are relevant for most models. Each procedure’s details and gate settings can be adjusted at each facility to fulfill the acceptance criteria for the PIGRET analysis.

#### Cytometer startup and plot creation

Start up the FCM as described in the instruction manual. Carry out the instrument’s quality control of the instrument as determined at each facility to ensure that it is optimal. Keep the flow cell and fluid lines clean to prevent detecting nonspecific events in each region.

**Fig. 2:**
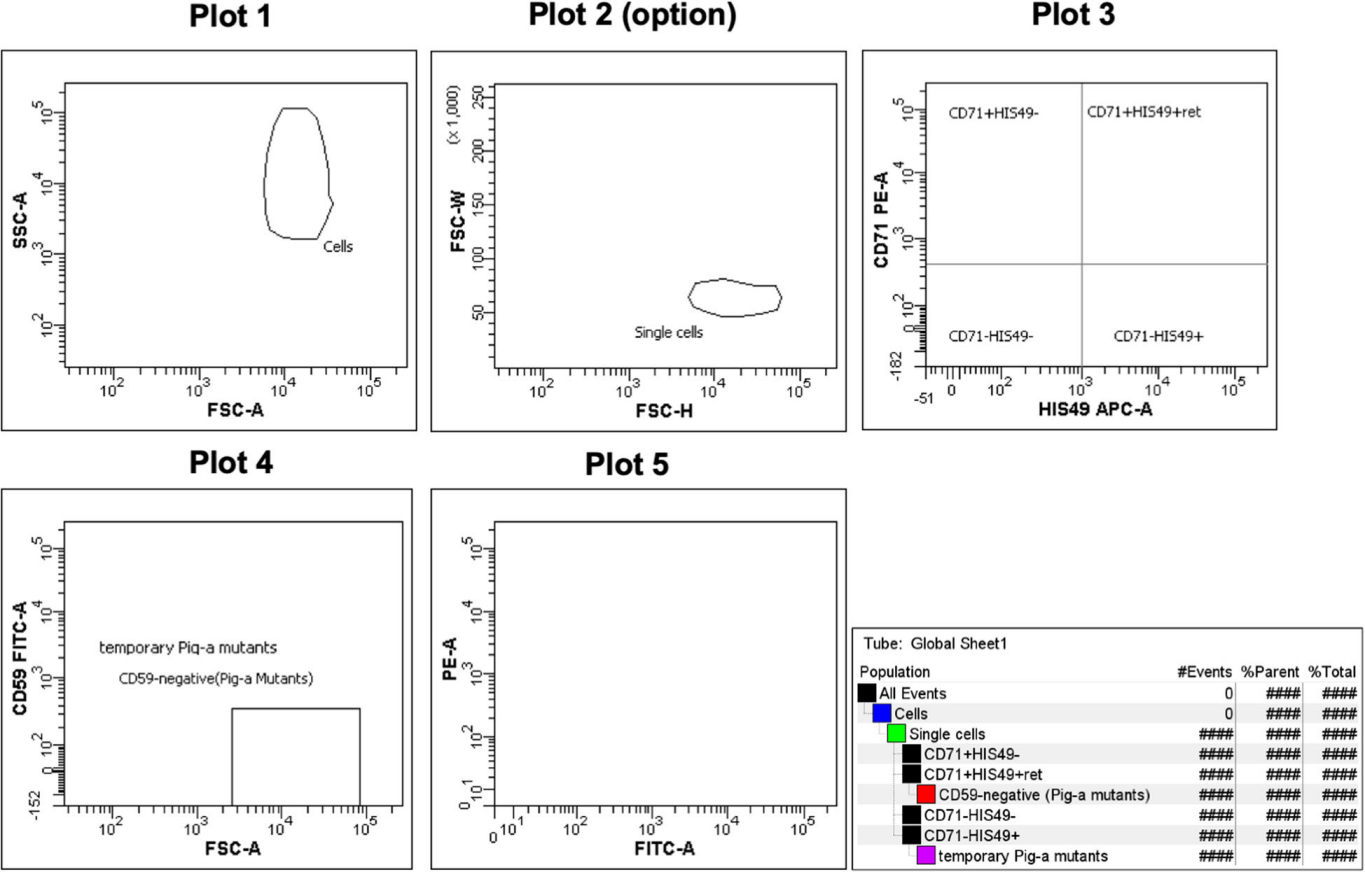
Plots for the PIGRET assay. Create dot-plots of FSC vs. SSC (Plot 1), PE vs. APC (Plot 3), FSC vs. FITC (Plot 4), and PE vs. FITC (Plot 5). Create one more dot-plot of FSC-H/FSC-W to eliminate doublet cells (Plot 2, option for digital FCM). Adjust or create the “*Pig-a* mutants” region to include 99.0% ± 0.1% of the CD59-negative cell population using the HIS49 single-stain sample

Create a worksheet and plots as shown in Fig. 2: dot-plots of forward scatter (FSC) vs. side scatter (SSC), PE vs. APC, FSC vs. FITC, and PE vs. FITC. Create one more dot-plot of FSC-H/FSC-W to eliminate the doublet cells. Using analysis software, such as FACSDiva, set the area scaling factor to an appropriate value so that the results of the analyses using area are were accurate. Analog instruments, such as FACSCalibur, do not have this function. Therefore, an area scaling factor and the FSC-H/FSC-W plot are not used.

During the loading of the non-stained sample or each single-stained sample, adjust the photomultiplier tube (PMT) voltages for all fluorescence parameters to be the negative or positive population plotted to an appropriate position. Adjust each negative population to be plotted at 10^3^ or below and each positive population to be at around 10^4^ on the axis. After adjusting the PMT voltages, calculate and apply compensation values for PE and FITC channels because PE and FITC fluorescence overlap into each other’s channel. Check a cell population on Plot-5 and confirm to apply the compensation values appropriately.

#### Creating gates with the non-stained sample

Place the non-stained sample on the FCM and acquire data (without recording). During acquisition, adjust the PMT voltage so that the cell population is plotted in the upper right quadrant of FSC/SSC, as shown in Plot 1 in Fig. 2. Create a “Cells” gate by enclosing the cell population in Plot 1 using a polygon or a freeform gate tool. If applicable, create a “Single cells” gate in Plot 2 in the same manner as “Cells,” and set a threshold option on FSC and SSC.

**Fig. 3:**
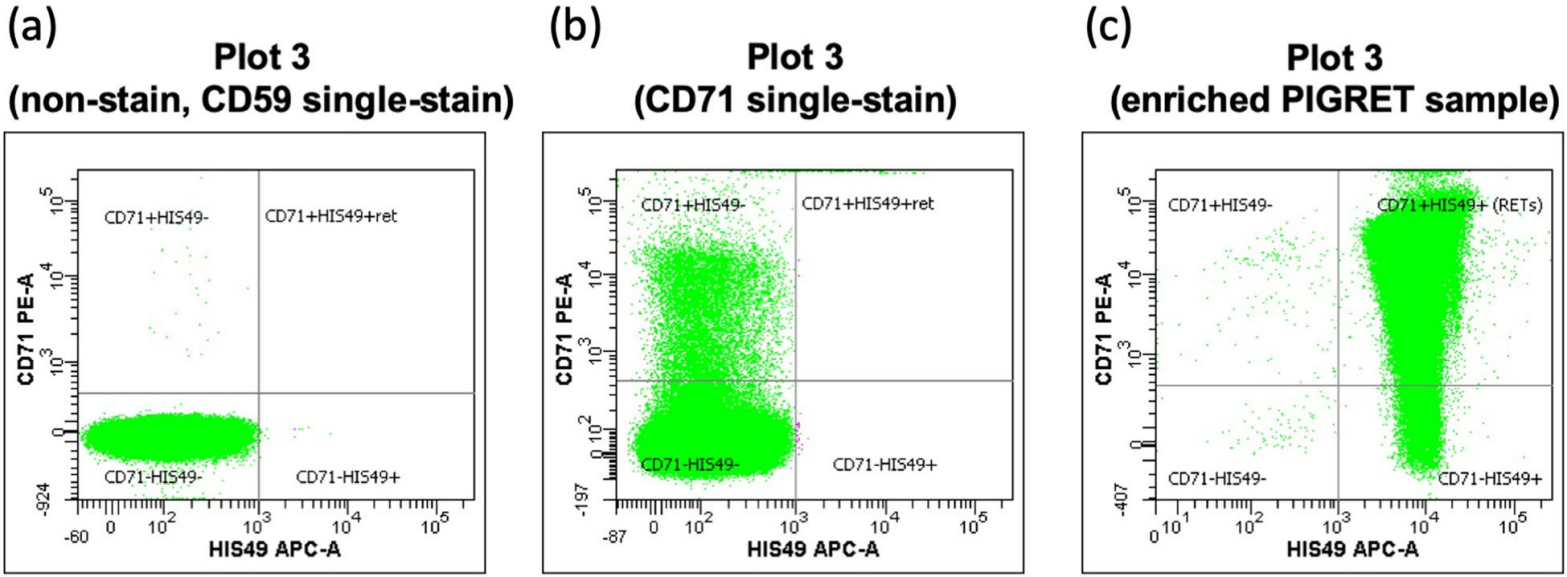
Typical images of PE vs. APC plot (Plot 3). **a** Typical plot for a non-stained sample and a CD59 single-stain sample, **b** plot for a CD71 single-stain sample, and (**c**) plot for PIGRET samples stained with anti-CD71, anti-CD59, and anti-HIS49 Abs

Then, create a quadrant gate in Plot 3, the dot-plot for PE vs. APC. Make sure that the quadrant gate is a subset of the “Single cells” gate, and the “Cells” population is a parent of the “Single cells” gate in the Population Hierarchy view. Adjust the quadrant gate in Plot 3 such that nearly all cells of the unstained cell sample (%parent value 99.0–100.0) are plotted within the lower left region: “CD71 − HIS49−” (Fig. 3a).

#### Defining gates for the *Pig-a* mutants with HIS49 single-stain samples

Create a “*Pig-a* mutants” gate indented below the region CD71 + HIS49+ and optimize the gate following the rule set in a previous collaborative study, the JHSF study [1]. Adjust the “*Pig-a* mutants” region to include 99.0% ± 0.1% of the CD59-negative cell population using the HIS49 single-stain sample. This step is critical for the accurate detection of *Pig-a* mutants but varied depending on the FCM model. The representative methods are described below (1) or (2). Once the PMT voltages and gates were set appropriately and saved as a protocol, subsequent analyses can be performed. Before the data acquisition with the saved protocol, fine-tune the PMT voltages and the gate settings. Make sure that the rule for the “*Pig-a* mutants” gate has been strictly applied to the setting, that is, the setting region 99.0% ± 0.1% of the CD59-negative cell population (“CD71 − HIS49+”) using the HIS49 single-stain sample.

1. Method using the FACSDiva software or equivalent (gate settings can be copied and pasted)

**Fig. 4:**
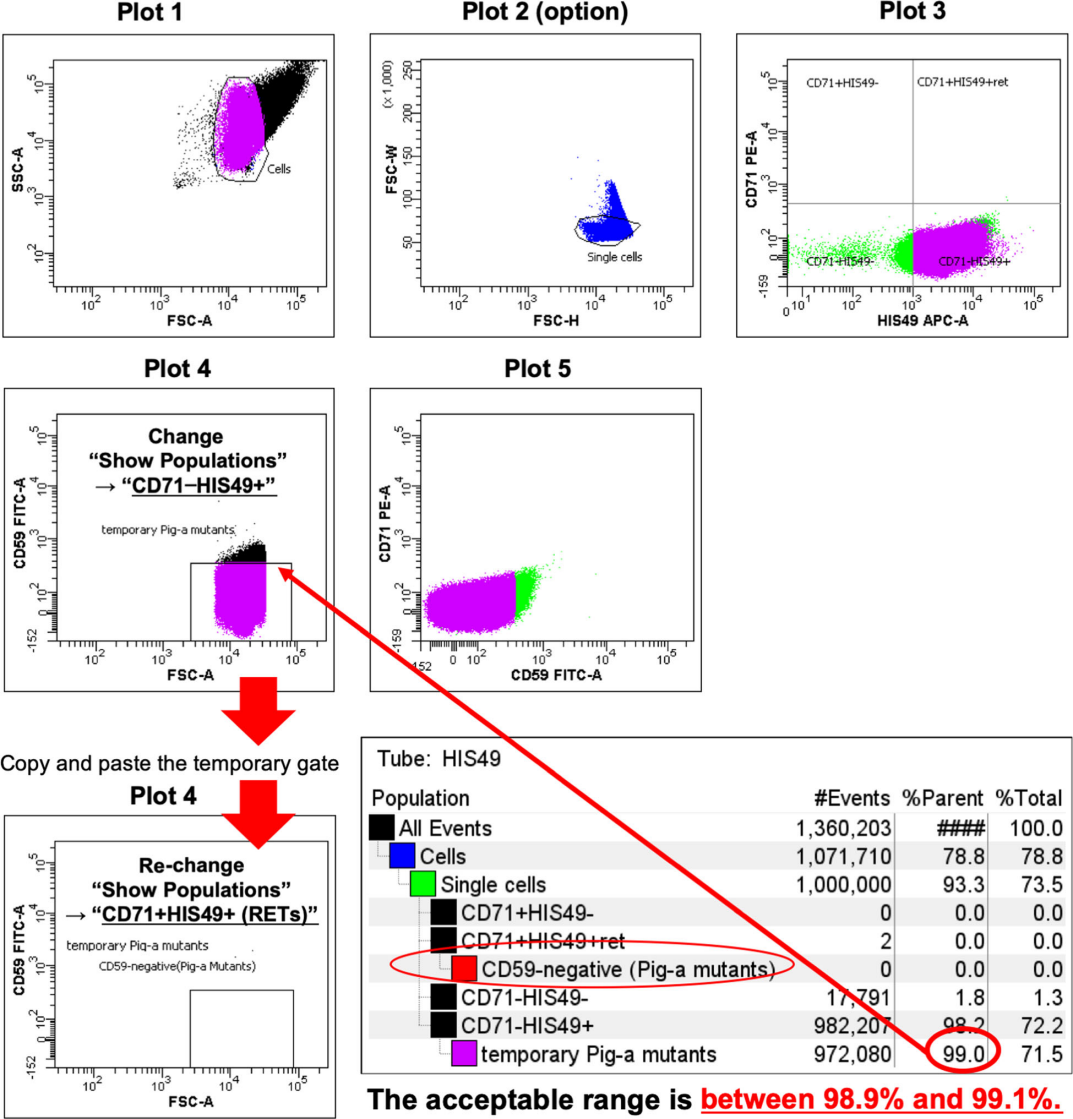
Gate defining and adjustment for *Pig-a* mutants with HIS49 single-stain samples (copy-and-paste method for FACSCantoII). Create “temporary *Pig-a* mutants” gate in Plot 4. Adjust the height of the “temporary *Pig-a* mutants” gate to include 99.0% ± 0.1% of the CD59-negative cell population using the HIS49 single-stain sample. Make sure that the “temporary *Pig-a* mutants” gate has reached the x-axis. If applicable, select “Bi-exponential” for the y-axis (FITC intensity scaling) in the Plot Inspector. Then, copy-paste the temporary gate so that the gate would be created as a subset of “CD71 + HIS49 + .” Rename to “*Pig-a* Mutants” and change Show Populations back to the “CD71 + HIS49+” population. The marker of “temporary *Pig-a* mutants” can be hidden if appropriate

Change a population displayed in Plot 4 so that “CD71 − HIS49+” cells will be displayed as the HIS49 single-stain sample is used. Create a “temporary *Pig-a* mutants” gate as a subset of the CD71 − HIS49+ region in Plot 4, as shown in Fig. 4. Acquire data for the HIS49 single-stain sample and adjust the “temporary *Pig-a* mutants” gate to include 99.0% (the acceptable range is between 98.9 and 99.1%) of the CD71-HIS49+ cell population. Confirm that the “temporary *Pig-a* mutants” gate has reached the x-axis.

Next, “temporary *Pig-a* mutants” in the Population Hierarchy view was selected, and the gate was copied. Then select the CD71 + HIS49+ region in the Population Hierarchy view and paste a gate, so that it would be created as a subset of “CD71 + HIS49+” and overlapped the “temporary *Pig-a* mutants” gate in Plot 4. Rename this new gate as the “*Pig-a* mutants” gate (Fig. 4). If applicable, select “Bi-exponential” for the y-axis (FITC intensity scaling) in the Plot Inspector. Change a population displayed in Plot 4 back to the “CD71 + HIS49+” cells.

(2). Method using FACS CellQuest or equivalent (numerical values of the gate settings can be visualized)

A “*Pig-a* mutants” gate on Plot 4 was created as a subset of “CD71 + HIS49+,” as shown in Fig. 4. Then, create another dot-plot same as Plot 4, name it Plot 4′, and draw and a rectangle gate in Plot 4′. Acquire data for the HIS49 single-stain sample and adjust the rectangle gate to enclose 99.0% (acceptable range is between 98.9 and 99.1%) of the “CD71-HIS49+” cell population. Check the Statistic view and set the max/min values of height for the “*Pig-a* mutants” gate as same as those for the rectangle gate in Plot 4′.

#### Check gate setting using the CD59 or CD71 single-stain sample

**Fig. 5:**
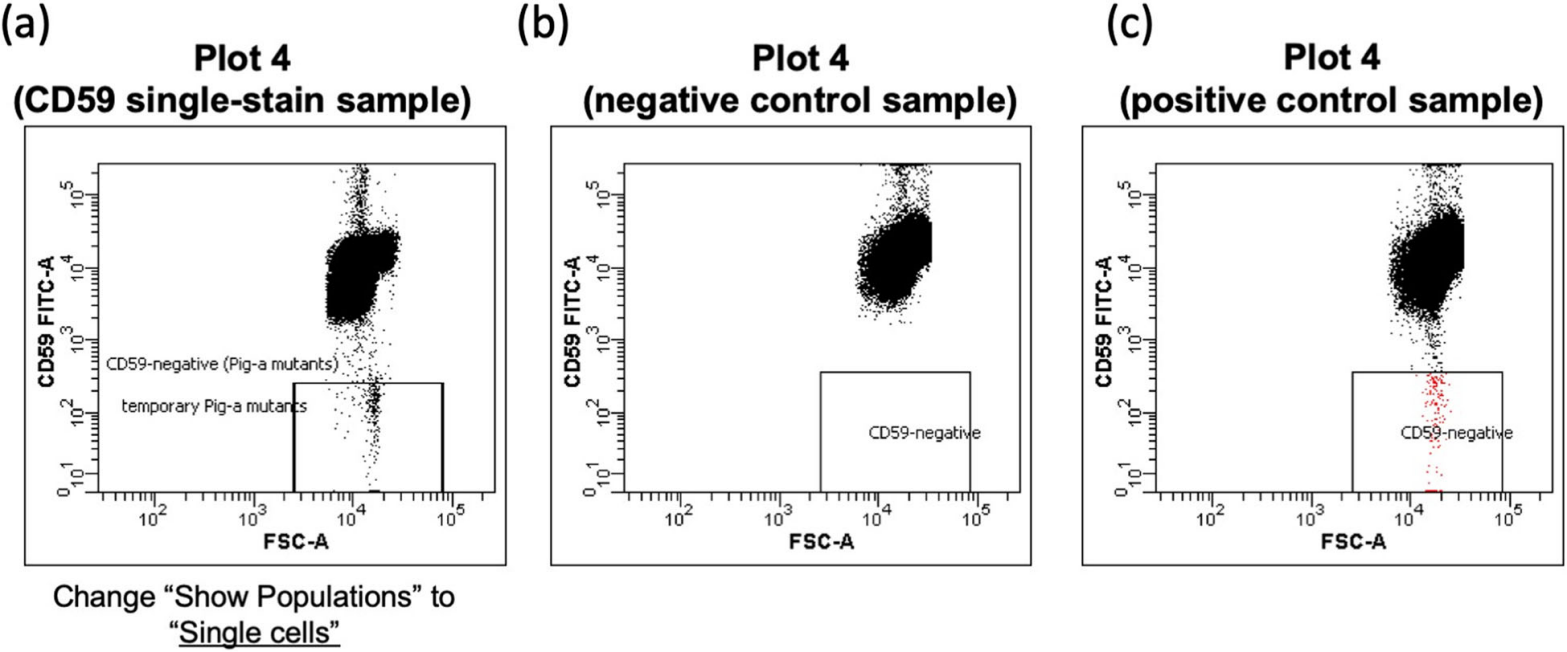
Typical images of the FSC vs. CD59-FITC plot (Plot 4). **a** Typical plot for a CD59 single-stain sample (Show Populations is set to “Single Cells”), **b** plot for a negative control sample, and (**c**) plot for a positive control sample treated with ENU 40 mg/kg by gavage

Data for the CD59 single-stain sample were acquired. Most cells in the “Single Cells” population will be plotted on the FITC-positive region in Plot 5. Visually confirm that most cells are plotted at 10^3^ or above without overlapping with the “*Pig-a* mutants” region in Plot 4 (Fig. 5).

Place the CD71 single-stain sample on the FCM and acquire data. Confirm that the “Single Cells” population (or “Cells”) is plotted on the left side of Plot 3: “CD71 − HIS49−” and “CD71 + HIS49-” regions (Fig. 3).

### Data collection

#### Data collection for the PIGRET assay

Acquire and record data for all control samples. Count at least 1 million cells plotted in the “Single cells” region. If the single-cell population is not defined, count cells in the “Cells” gate instead. Ensure that each gate is set appropriately (particularly the “*Pig-a* mutants” region setting; 99.0% ± 0.1% rule defined above) while the non-stained or single-stain control samples are being recorded. Then, load PBS or purified water, and wash the fluid lines for several minutes before the analysis of the assay samples to prevent contamination of the non-stained cells.

Immediately before loading the assay sample on the FCM, thoroughly mix the sample by vortexing. Filter the sample solutions with a cell strainer if cell clumps are present. Load assay samples for the PIGRET assay on the FCM, and record data. It is advisable to collect and save all events to the database rather than certain cell populations (e.g., cells in the “Cells” region) so that the complete data are available for re-analysis if needed.

Count at least 1 million cells in the “CD71 + HIS49+” region: RETs. Maintain the sample flow rate at 10,000 events/s or below by adjusting the event rate on the instrument or the cell concentration of each sample to reduce nonspecific data and conduct an efficient sample analysis. If many cells are detected in the “*Pig-a* mutants” region (Fig. 5c), load PBS or purified water to wash the fluid lines. After washing, acquire the data of the next samples’ data and confirm its plot, and then continue recording the data.

#### Analysis of %RETs

The %RETs is also analyzed using the same protocol file for the PIGRET assay, excluding Plot 4. Confirm the region settings, acquire data for at least 10,000 or above cells in “Single cells” or “Cells” for the three control samples prepared for the PIGRET assay: non-stained sample, the HIS49 single-stain sample, and the CD71 single-stain sample.

**Fig. 6:**
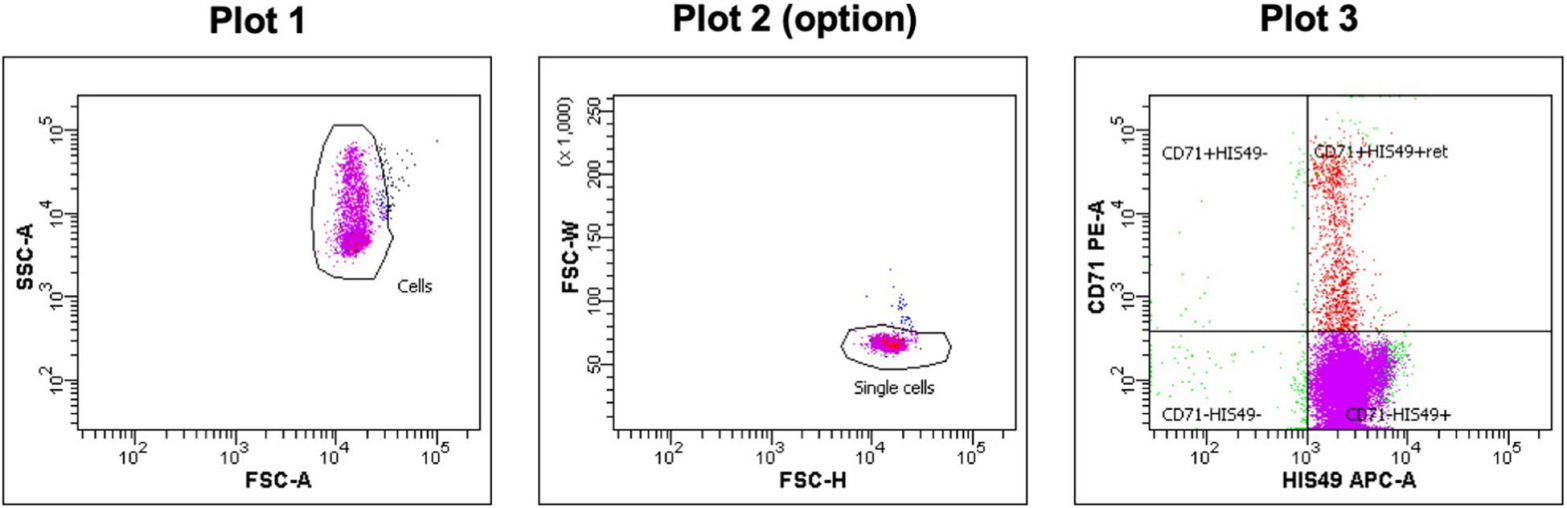
Example of plots for %RET. Use same plots for the PIGRET assay (plots 1–3). If a gate setting, especially the quadrant gate for Plot 3, was adjusted in the PIGRET assay, make sure to use same gate setting for %RET as PIGRET assay

Then, place assay samples for %RETs on the FCM, and record data for at least 10,000 cells in the “CD71-HIS49+” region (Fig. 6). The analysis for %RETs can be performed at a much lower sample flow rate than the PIGRET assay and completed quickly.

### Data analysis

#### Calculation of the *Pig-a* MF

The *Pig-a* MF is calculated according to the following formula:

$$ Pig-a\  MF\ \left({10}^{-6}\right)=\frac{\mathrm{Number}\ \mathrm{of}\ \mathrm{CD}59\ \mathrm{negative}\ \mathrm{RETs}\ \left(`` Pig-a\ \mathrm{mutants}"\right)}{\mathrm{Number}\ \mathrm{of}\ \mathrm{RETs}\ \left(``\mathrm{CD}71+\mathrm{HIS}49+"\right)}\times {10}^6 $$

#### Calculation of %RETs

The %RETs is calculated according to the following formula (when using the FCM):

$$ \%\mathrm{RETs}=\frac{\mathrm{Number}\ \mathrm{of}\ \mathrm{RETs}\ \left(``\mathrm{CD}71+\mathrm{HIS}49+"\right)}{\begin{array}{c}\mathrm{Total}\ \mathrm{number}\ \mathrm{of}\ \mathrm{RBCs}\\ {}\left(``\mathrm{CD}71+\mathrm{HIS}49+"+``\mathrm{CD}71-\mathrm{HIS}49+"\right)\end{array}}\times 100 $$

#### Acceptance criteria for the assay

The negative/vehicle and positive control groups are required to satisfy the criteria below [see Note (e)]:

- The average *Pig-a* MF (10^− 6^) in the negative/vehicle control group is 5 or below.
- The *Pig-a* MF (10^− 6^) of each animal in the positive control group is significantly different from that in the negative control group (10 or above is adequate).

### Statistical analysis

In the MMS/JEMS collaborative study, statistical analyses of *Pig-a* MF data were performed following the IWGT report’s method.

An offset of 0.1 is added to each *Pig-a* MF value (expressed as mutants × 10^−6^ RETs) in case that *Pig-a* MF values of zero are occasionally observed, and the values are log_10_ transformed. To avoid the “zero value,” however, there is another way to count RETs until the first mutant cell is observed so that a statistical analysis can be performed without adding 0.1. Then, transformed *Pig-a* MF values are analyzed by Bartlett’s test for homogeneity of variance among the groups. If the group variance is determined to be homogeneous, the significance of increases in treated rats relative to negative control groups is analyzed using Dunnett’s multiple comparison test. If Bartlett’s test indicates heterogeneous variance, the nonparametric Dunnett’s multiple comparison test (Steel test) is used. Significance is evaluated at the 5% level using a one-tailed test for increases relative to the negative or vehicle control.

The %RETs are analyzed using Bartlett’s test for homogeneity of variance among the groups. Subsequently, a parametric or nonparametric Dunnett’s multiple comparison test is employed. Significance is evaluated at the 5% level using a two-tailed test relative to the vehicle control.

When the acceptance criteria for the assay were fulfilled, a test chemical was judged statistically positive or negative if it met the following criteria in our collaborative study [see Note (f)].

- Positive

A treated group shows a statistically significant increase in the *Pig-a* MF as compared with the concurrent negative/vehicle control.

- Negative

There is no statistically significant increase in the *Pig-a* MFs in any sampling points of treated groups.

### Tips for conducting the PIGRET assay

Increased hematopoiesis does not affect evaluating the mutagenic potential of chemicals in the *Pig-a* assays [34, 35]. A bone marrow toxicity by test chemical may make it difficult to conduct the assay with RETs due to the reduction of absolute number in whole blood. A sufficient recovery time after the cessation or an inclusion of lower dose level without severe bone marrow toxicity may help the evaluation of in vivo mutagenicity using the PIGRET assay.

**Fig. 7:**
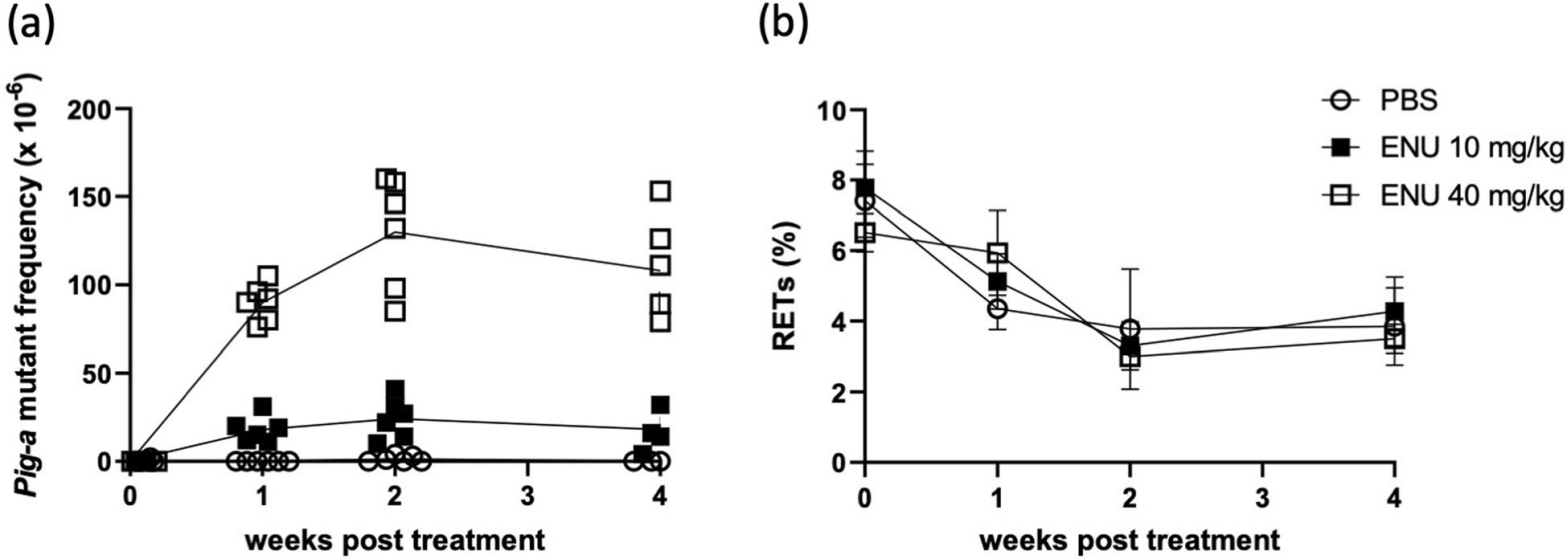
Example of PIGRET assay results. **a** PIGRET assays were conducted at 0 (pretreatment), 1, 2, and 4 weeks after a single oral administration of PBS (negative control; open circles), 10 mg/kg ENU (filled squares), or 40 mg/kg ENU (open squares). **b** %RET analysis results at 0, 1, 2, and 4 weeks. This study was conducted at Teijin Pharma Limited, and the result of PIGRET assay was reported as the reference site response for the MMS/JEMS collaborative study (Step 1) [2]. The %RET data have not been previously published

When each facility establishes and validates the PIGRET assay techniques on-site, it is desirable to conduct an assay on rats treated with a single dose of 10 and 40 mg/kg ENU by gavage and confirm that the *Pig-a* MF increases significantly at 2 or 4 weeks after administration in 10 mg/kg ENU-treated group (Fig. 7a). The average *Pig-a* MF in the negative control group should be consistently 5 × 10^− 6^ or below.

To evaluate compounds correctly, ensure that the PMT voltages are adjusted appropriately and that the CD59-positive (wild-type) population has sufficient FITC intensity. If normal (wild-type) cells have insufficient fluorescence intensity despite the PMT voltage adjustment, it is better to change the Ab. Keep the flow cell and fluid lines clean to prevent the detection of nonspecific particles.

### Notes

- The RET ratio decreases with age (Fig. 7b). At age ~ 8 weeks, a sufficient number of RETs for the PIGRET assay can be obtained from 80 μL whole blood. When using aged animals, the number of enriched RETs decreases, and 10^6^ cells cannot be counted. To maintain an effective throughput performance, increase the volume of whole blood to about 150 μL depending on the week’s age. When bone marrow suppression is a concern, increase volume up to 200 μL and mix with prescribed PE-CD71 Ab solution.
- EDTA and heparin can be used as anticoagulants for the *Pig-a* assays for preservation for a few days. EDTA is better for long preservation because some blood samples with heparin coagulated during 7-day preservation in our laboratory [31].
- For example, if two commercially available magnet stands that can hold 6 samples are used, the immunomagnetic separation is performed in two to three batches as follows:
  Start the first batch procedure and then start the second batch during the 15-min incubation with the PE Magnetic Particles Plus-DM beads of the first batch. Start the third batch after the first batch separation has completed, and the second batch might be at the beads incubation step. Enriched samples can be stored in the refrigerator but should be used on the same day. The coagulation of the samples may cause false-positive results due to poor staining. It is advisable to remove the supernatant completely at each step and suspend with PBS well for an efficient and accurate assay.
- Using a single-laser FCM, choose PerCP-Cy5.5-labeled anti-erythroid marker Ab, which can be used with FITC and PE-labeled reagents in a single-laser FCM. Kikuzuki et al. conducted the *Pig-a* assay with Epics XL equipped with SySTEM II software in the MMS/JEMS collaborative study, and their results satisfied with acceptance criteria [28].
  PerCP-Cy5.5-conjugated anti-erythroid marker Ab (clone HIS49) was prepared. Then, add 0.8 μg HIS49 antibody per sample. If the PerCP-Cy5.5-conjugated HIS49 Ab stock solution is 0.2 mg/mL, the master mix is prepared by mixing 2 μL anti-CD59 Ab stock solution and 4 μL HIS49 Ab per sample for the total number of assay samples. Then, add 6 μL master mix to each sample tube. When analyzing %RETs, use 4 μL HIS49 Ab and 5 μL PE-CD71 Ab per sample.
  Create plots using PerCP-Cy5.5 instead of APC, and adjust the gates in the same manner. If needed, calculate and apply compensation values.
- Acceptance criteria in this protocol are intended for first-time users of the PIGRET assay.
  Positive control:
  A positive control group is required as an acceptance criterion to validate the *Pig-a* assay technique on-site. According to the IWGT workshop recommendations, a positive control group is not considered mandatory if an appropriate standard that “mimics” mutants is used each time for FCM analysis.
  Negative/vehicle control:
  After validating the *Pig-a* assay technique and gathering enough historical data on-site, the acceptance criteria can include comparing the negative control values to the historical negative control distribution for the laboratory.
- Preparations are now underway for the new OECD test guideline for the in vivo *Pig-a* assay, and the interpretation of results is a hotly debated issue. In case a test compound is judged statistically positive, the final judgment may be concluded taking into account the biological relevance, such as a dose-related increase with an appropriate trend test, time-course, and own historical control data limit.

## Conclusions

The *Pig-a* assay has attracted international attention, and the IWGT has discussed its potential for regulatory use. The preparation for the new OECD test guideline for the in vivo *Pig-a* assay is ongoing. The PIGRET assay has several desirable features: high-throughput assay system, higher sensitivity to mutagens, and early detection of mutation. The protocol described here was validated by the MMS/JEMS collaborative study, and the results were reported in a special issue of *Mutation Research* (Vol. 811, 2016) [2]. It is expected that studies using this protocol will provide important information for the further development of the *Pig-a* assay.

## Data Availability

Not applicable.

## Abbreviations

APC-HIS49 Ab: APC-conjugated anti-rat erythroid marker antibody
FCM: Flow cytometry
FITC-CD59 Ab: FITC-conjugated anti-rat CD59 antibody
FSC: Forward scatter
GPI: Glycosylphosphatidylinositol
IWGT: International Workshop on Genotoxicity Testing
JHSF: Japan Health Sciences Foundation
MF: Mutant frequency
OECD: Organisation for Economic Co-operation and Development
PE-CD71 Ab: Phycoerythrin-conjugated anti-rat CD71 antibody
PMT: Photomultiplier tube
RBC: Red blood cells
RET: Reticulocytes
SSC: Side scatter
